# An FCN1^+^ macrophage-associated SEMA3C–NRP1 axis is linked to fibroblast reprogramming in RA-ILD

**DOI:** 10.3389/fimmu.2026.1978823

**Published:** 2026-10-23

**Authors:** Ruofan Su, Licheng Song, Xuxin Chen, Yaru Liu, Yi Yang, Chunyang Zhang, Junjie Du, Dingyun Song, Li Xiao, Lixin Xie, Zhihai Han

**Affiliations:** 1 Senior Department of Respiratory and Critical Care Medicine, the Eighth Medical Center of PLA General Hospital, Beijing, China; 2 Medical School of Chinese PLA, Beijing, China; 3 Respiratory Research Institute, Department of Pulmonary & Critical Care Medicine, the Eighth Medical Center of PLA General Hospital, Beijing, China; 4 Beijing Key Laboratory of OTIR, Beijing, China

**Keywords:** lipofibroblast, macrophage, NRP1, rheumatoid arthritis–associated interstitial lung disease, SEMA3C, single-cell RNA sequencing

## Abstract

**Introduction:**

Rheumatoid arthritis–associated interstitial lung disease (RA-ILD) is a major extra-articular complication of rheumatoid arthritis and a leading cause of morbidity and mortality. Unlike idiopathic pulmonary fibrosis (IPF), RA-ILD develops in the context of systemic immune activation, but the cellular basis linking inflammation to fibroblast-state changes remains incompletely understood.

**Methods:**

We performed single-cell RNA sequencing on lung tissues from healthy donors, patients with RA-ILD, and patients with IPF, complemented by spatial validation and *in vitro* experiments.

**Results:**

In this cohort, RA-ILD lungs showed an increased proportion of FCN1⁺ inflammatory macrophages within the macrophage compartment, whereas IPF lungs were dominated by SPP1⁺ macrophages. Trajectory and cell–cell communication analyses identified an RA-ILD-enriched matrix-fibroblast-to-CXCR4⁺ lipofibroblast program and a predicted SEMA3C–NRP1 interaction between inflammatory macrophage populations and fibroblasts; these patterns were not detected in the IPF or healthy-control samples analyzed here. Multiplex immunofluorescence showed macrophage-associated SEMA3C signal in spatial proximity to NRP1⁺ fibroblasts within fibrotic regions. A flow-sorted inflammatory monocyte/macrophage-enriched population from RA-ILD lungs had higher supernatant SEMA3C levels and was associated with shifts in MRC-5 fibroblast markers from EBF1/PDGFRA toward APOE/CXCR4. Recombinant SEMA3C reproduced this marker-defined shift, whereas pharmacological NRP1 inhibition attenuated it; an orthogonal THP-1 overexpression model produced concordant results.

**Discussion:**

These data support an association between an inflammatory macrophage–SEMA3C–NRP1 axis and fibroblast-state remodeling in the studied RA-ILD samples. Because the RA-ILD cohort was small and did not include other CTD-ILD subtypes, the findings should not be interpreted as establishing RA-ILD specificity or a fully profibrotic functional phenotype.

## 1 Introduction

Fibrotic interstitial lung diseases (ILDs) are progressive conditions characterized by high morbidity and mortality, ultimately leading to pulmonary fibrosis (1, 2). Among them, rheumatoid arthritis–associated interstitial lung disease (RA-ILD) is one of the most clinically important forms of connective tissue disease–associated ILD (CTD-ILD) and represents a major cause of death in patients with rheumatoid arthritis (3, 4). RA-ILD is typically managed with a combination therapy involving long-term immunosuppressive therapy combined with short-term glucocorticoids, supplemented with nintedanib when necessary. In contrast, treatment for idiopathic pulmonary fibrosis (IPF) primarily involves antifibrotic drugs such as pirfenidone and nintedanib. Although both diseases ultimately result in pulmonary fibrosis, the molecular mechanisms underlying fibrosis in RA-ILD remain unclear compared to the extensively studied IPF. It is still uncertain whether RA-ILD possesses unique fibrosis pathways.

Macrophages and fibroblasts are central regulators of tissue injury, repair, and fibrotic remodeling (5–7). Recent single-cell RNA sequencing studies of human fibrotic lungs have revealed marked heterogeneity within the macrophage compartment. Among these subsets, SPP1^+^ macrophages have been strongly linked to fibrotic remodeling in IPF, whereas FABP4^+^ macrophages are enriched in healthy lungs. In addition, FCN1^+^ inflammatory macrophages have emerged as a distinct monocyte-derived population associated with inflammatory tissue responses (8). Nevertheless, whether specific macrophage subsets contribute to fibroblast activation and disease-specific fibrotic remodeling in RA-ILD remains unclear.

Macrophages and fibroblasts engage in complex crosstalk that forms a profibrotic macrophage–fibroblast axis. *In vitro* studies have shown that macrophage-conditioned media directly promote fibroblast proliferation and extracellular matrix (ECM) production (9, 10). Moreover, SPP1+ macrophages have been shown to play a critical role in activating myofibroblasts and promoting fibrosis not only in the lungs of IPF patients, but also in the kidneys and liver (6, 8, 11). However, the specific macrophage subsets involved in fibrotic progression in RA-ILD, and how they interact with fibroblasts—the major collagen-producing effector cells driving fibrotic remodeling—remain poorly understood. Semaphorin 3C (SEMA3C) is a secreted member of the class 3 semaphorin family that signals through receptor complexes containing neuropilins and plexins to regulate cell migration and intercellular communication (12). In addition to its established roles in developmental guidance and cancer, SEMA3C has been implicated in fibrotic remodeling, including the enhancement of TGF-β-dependent myofibroblast activation in liver fibrosis (13–15). However, whether SEMA3C mediates communication between inflammatory macrophages and fibroblasts in RA-ILD remains unknown.

To address this question, we performed single-cell RNA sequencing on lung tissue samples from healthy donors, patients with RA-ILD, and patients with IPF. We observed distinct distribution patterns of macrophage subpopulations across the three groups. FABP4^+^ macrophages predominated in healthy lungs, whereas SPP1^+^ macrophages were enriched in IPF lungs, consistent with previous studies. In the RA-ILD samples analyzed here, FCN1^+^ macrophages constituted the largest macrophage subset. CellChat analysis further predicted an interaction between inflammatory macrophage populations and fibroblasts through the SEMA3C–NRP1 axis that was detected in RA-ILD but not in the IPF or healthy-control samples in this dataset. *In vitro* experiments supported SEMA3C–NRP1-dependent changes in fibroblast-state markers. In this study, “lipofibroblast” refers to an APOE-expressing fibroblast cluster identified by single-cell transcriptomic analysis, rather than functionally validated canonical PPARγ^+^ lipid-storing lipofibroblasts. Thus, this study identifies a candidate inflammation-associated macrophage–fibroblast pathway in RA-ILD.

## 2 Materials and methods

### 2.1 Inclusion and exclusion criteria

Patients were included if they had a confirmed diagnosis of rheumatoid arthritis–associated interstitial lung disease based on clinical, radiological, and multidisciplinary evaluation. Patients with active infection, malignancy, or incomplete clinical information were excluded. Both male and female patients were included in this study. Sex was recorded for all subjects, but no sex-specific analyses were performed. Demographic information, including age and sex, was collected for all participants and is summarized in **Figure 1b**. Randomization was not applicable because this was an observational study of human tissue samples and *in vitro* mechanistic experiments.

**Figure 1:**
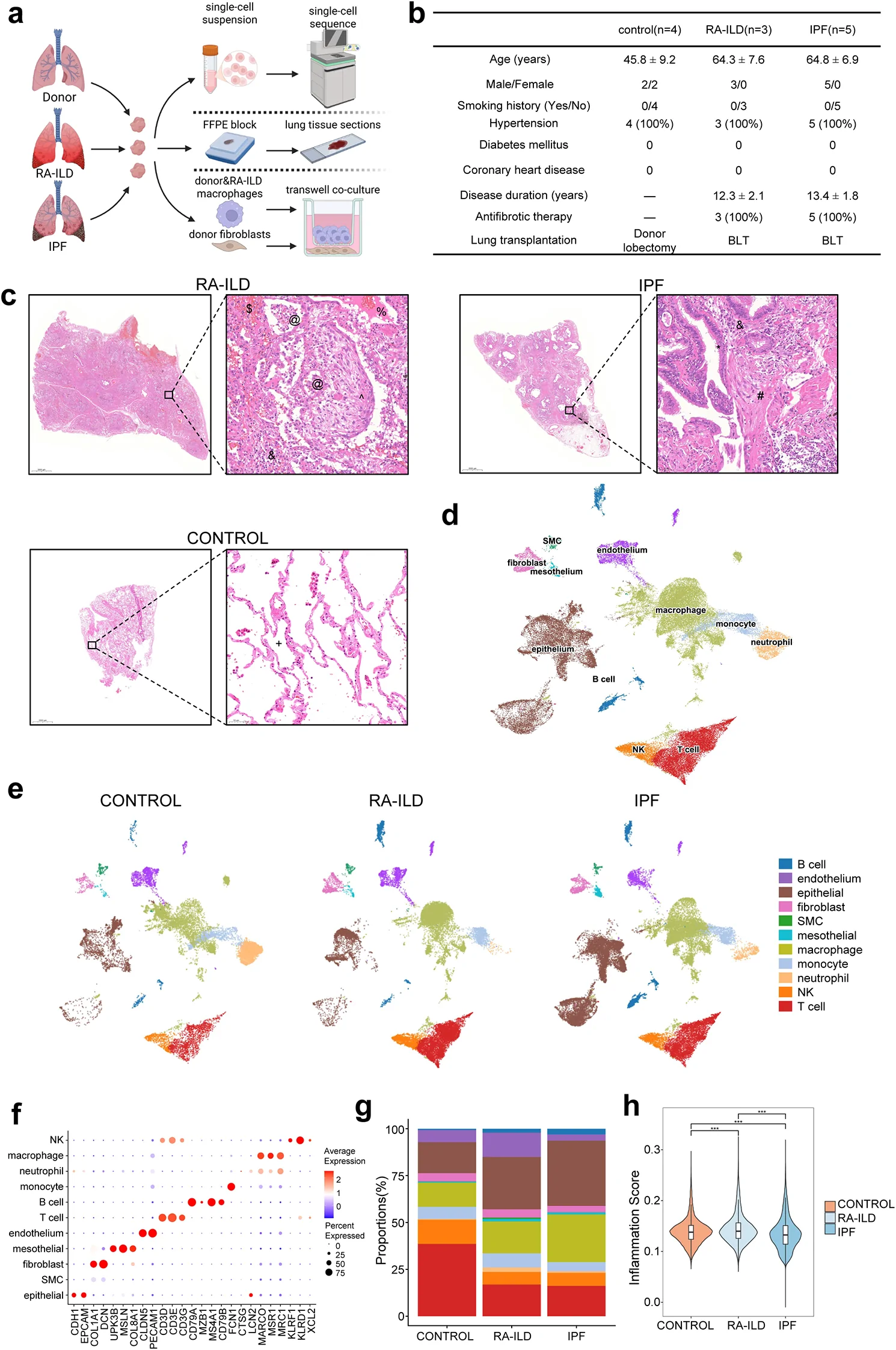
Single-cell transcriptomic profiling and histological characterization of lung tissues from HCs, RA-ILD, and IPF patients. **(a)** Schematic representation of the study design. Lung tissues from healthy donors, RA-ILD patients, and IPF patients were processed into FFPE blocks and fresh sections for histological evaluation and single-cell RNA sequencing. An inflammatory monocyte/macrophage-enriched fraction isolated from lung tissue and MRC-5 fibroblasts were used in complementary *in vitro* experiments. **(b)** Clinical characteristics of sequenced samples, including patient age, sex, diagnosis, disease etiology, and lung transplantation site. **(c)** Representative H&E staining images illustrating histopathological alterations. RA-ILD and IPF lungs displayed distinct lesions, including fibroblast foci (#), epithelial metaplasia (*), immune cell aggregates (&), alveolar hemorrhage ($), macrophage proliferation and clustering (@), fibroblast accumulation (^), and alveolar edema (%), whereas HCs lungs preserved normal alveolar architecture (+). **(d)** UMAP projection of all integrated single-cell transcriptomes colored by major cell lineages (epithelial, fibroblast, endothelial, and immune populations). **(e)** UMAP plots separated by group (HCs, RA-ILD, IPF), demonstrating marked shifts in cellular composition and distribution under fibrotic conditions. **(f)** Dot plot of canonical marker gene expression confirming cluster annotation. **(g)** Stacked bar plots showing the proportional changes in major cell populations across groups. **(h)** Violin plots showing cell-level inflammation-score distributions in the control, RA-ILD, and IPF groups. Each violin represents the distribution of scores calculated for individual cells pooled from the corresponding study group.

### 2.2 Clinical data collection and ILD pattern classification

Clinical information, including glucocorticoid use, antifibrotic treatment, and treatment status at the time of tissue collection, was retrospectively obtained from the medical records. Patient-level clinical and treatment information is summarized in **Supplementary Table 1**. Chest high-resolution computed tomography (HRCT) images were reviewed by two thoracic radiologists/two pulmonologists who were blinded to the single-cell and experimental results. Radiological patterns were classified as UIP, probable UIP, indeterminate for UIP, NSIP, OP, or other patterns. Histopathological patterns were independently evaluated by two pulmonary pathologists using the available surgical lung tissue sections. Disagreements were resolved by consensus. The radiological and histopathological patterns for individual patients are provided in **Supplementary Table 1**.

### 2.3 Lung tissues collection

Human lung tissue samples were divided into three groups: healthy controls (HCs, n = 4), patients with rheumatoid arthritis–associated interstitial lung disease (RA-ILD, n = 3), and patients with idiopathic pulmonary fibrosis (IPF, n = 5). Lung tissues were obtained either from patients undergoing clinically indicated procedures or from unused donor tissue during lung transplantation.

This study was approved by the Medical Ethics Committee of the Chinese PLA General Hospital (No. 2022052701006). Written informed consent was obtained from all participants, or from their legally authorized representatives when applicable, for tissue collection and the use of protected health information (PHI).

Lung tissues were washed with ice-cold PBS, minced into approximately 0.2–0.6 mm³ fragments, and enzymatically digested in a digestion solution containing dispase, collagenase, elastase, and DNase at 37 °C. Digestion was terminated by adding serum-containing medium, followed by mechanical dissociation, trituration, filtration, and red blood cell lysis when necessary. The resulting single-cell suspensions were filtered through 70-μm and 40-μm strainers before loading onto the 10x Genomics Chromium platform. Single-cell RNA-seq libraries were prepared using the Chromium Single Cell 5′ Library & Gel Bead Kit v2 (10x Genomics, Pleasanton, CA, USA; Cat. No. PN-1000263) according to the manufacturer’s instructions and the 10x Genomics Cell Preparation Guide. For each sample, approximately 10,000 cells were loaded onto the Chromium platform. Libraries were sequenced on an Illumina NovaSeq 6000 platform to a depth of approximately 50,000 reads per cell. Raw sequencing data were processed using Cell Ranger v4.0.0. Reads were aligned to the human reference genome GRCh38, and gene–cell count matrices were generated using the Cell Ranger count pipeline.

No animal experiments were performed in this study.

### 2.4 Single-cell RNA-seq data processing and analysis

The expression count matrix was analyzed using the Seurat v5.0.3 R package (https://satijalab.org/seurat). Cells were quality-controlled by retaining those with at least 300 detected genes and a mitochondrial transcript fraction of no more than 20%.

Potential doublets were identified independently for each donor using Scrublet (version 0.2.3). Scrublet was applied after initial quality-control filtering. Running the analysis separately for each donor avoided the generation of artificial cross-donor doublets. Scrublet was run with an expected doublet rate of 0.06 and a fixed random seed of 20250913. The parameters used for doublet simulation and scoring were min_counts = 2, min_cells = 3, min_gene_variability_pctl = 85, and n_prin_comps = 30. Because the automatically estimated Scrublet thresholds were unstable in several samples, particularly in samples without a clearly separated simulated-doublet score distribution, we applied a consistent rank-based thresholding rule before downstream clustering and annotation. For each donor, the doublet-score threshold was defined as the 94th percentile of the observed Scrublet score distribution, corresponding to the prespecified expected doublet rate of approximately 6%. Cells with scores greater than or equal to the donor-specific threshold were excluded.

The FindVariableFeatures function was used to identify genes with the greatest expression variability in the single-cell RNA-seq data. Each gene was then mean-centered and standardized, and the mean and standard deviation of each gene were computed for subsequent PCA dimensionality reduction. To mitigate inter-individual technical variation, individual donor/patient identity was specified as the batch variable in Harmony (16). All libraries were prepared and sequenced within the same sequencing run using the same protocol and flow cell, minimizing sequencing batch effects. Lung tissue sampling was performed from anatomically comparable regions. The FindClusters function was used to group cells into clusters, with the resolution parameter set to 0.8. Cell distributions were visualized in two-dimensional space using Uniform Manifold Approximation and Projection (UMAP).

### 2.5 Myeloid marker, module-score, and doublet analyses

To further validate myeloid cell annotation, predefined marker panels were used for neutrophils/granulocytes (FCGR3B, CSF3R, CXCR2, MPO, CEACAM8, S100A8, S100A9, S100A12, and FPR1), monocytes (FCN1, VCAM, CCR2, FCGR3A, LST1, CTSS, FCGR1A, and TYMP), and macrophages (C1QA, C1QB, C1QC, APOE, MARCO, MRC1, FABP4, PPARG, SPP1, and CD68). Lineage-specific module scores were calculated from the log-normalized expression matrix using Scanpy score_genes, with control gene sets containing at least 25 genes.

Potential neutrophil–macrophage mixed signals were assessed using raw UMI counts. Neutrophil-core breadth was defined as the number of detected genes among FCGR3B, CSF3R, CXCR2, and MPO, whereas macrophage-core breadth was defined as the number of detected genes among nine macrophage markers (C1QA, C1QB, C1QC, APOE, MARCO, MRC1, FABP4, PPARG, and CD68). Residual doublet risk was further evaluated in the final myeloid compartment using the retained Scrublet doublet scores, the original automated and donor-specific controlled doublet calls, UMI and gene-detection complexity, and the spatial distribution of doublet scores on the myeloid UMAP. In addition, clusters showing discordant expression of markers from otherwise unrelated lineages were inspected as potential cross-lineage doublets. The median residual doublet score was 0.0440. For visualization of the residual high-score tail only, cells above the global 99th percentile of the retained myeloid-cell doublet-score distribution (0.1153) were highlighted. This cutoff was not used for doublet removal.

### 2.6 Trajectory analysis

Cell trajectory analysis was performed using cells from all three study groups (HC, RA-ILD, and IPF) collectively with Monocle 2 (version 2.28.0). Quality-controlled and normalized single-cell RNA-sequencing data were imported into Monocle to generate a CellDataSet object. Genes showing high cell-to-cell variability were selected for trajectory construction. Dimensionality reduction was performed using the DDRTree algorithm implemented in the reduceDimension function. Cells were visualized in the reduced space and annotated based on established marker genes. Pseudotime ordering was performed using the orderCells function, with biologically relevant cell populations chosen as the root state. Gene expression changes along pseudotime were examined to characterize transitions between cell states.

### 2.7 Cell-cell interaction analysis

CellChat was used to deduce ligand-receptor interactions (v1.6.1, https://github.com/jinworks/CellChat) (17). Genes were retained if they were expressed in at least 10% of cells within the relevant cluster and were identified as overexpressed genes using the Wilcoxon test with Benjamini–Hochberg-adjusted FDR < 0.05. Interaction probabilities were computed using computeCommunProb with 500 permutations and a truncated mean approach with trim = 0.1. Raw p values from permutation were adjusted using the Benjamini–Hochberg method; ligand–receptor pairs with FDR < 0.05, probability ≥ 0.05, and ligand/receptor expression fraction ≥ 0.1 in the sending and receiving clusters were retained. Pathway-level scores were calculated by aggregating significant ligand–receptor pairs; only pathways with ≥50% constituent pairs meeting the above criteria were interpreted. Consistency across patients was assessed by repeating the analysis on per-patient pseudobulk cell groups and retaining interactions present in at least half of the individuals.

### 2.8 Functional scoring analysis

We used the UCell package to calculate functional scores for predefined gene modules related to inflammation and tissue repair (chronic inflammatory response, GO:0002544). Prior to functional gene set scoring, we removed housekeeping genes with high expression from each gene set to prevent bias introduced by their constitutive expression. Marker genes for each module were curated, and the AddModuleScore_UCell function was applied to calculate functional scores for individual cells. These cell-level scores were extracted using FetchData and visualized across groups (healthy controls, IPF, and RA-ILD) using violin and box plots generated with ggplot2.

### 2.9 Multiplex immunofluorescence staining of human lung tissues

For immunofluorescence validation, paraffin-embedded lung tissues from five independent subjects in each group (HC, RA-ILD, and IPF; n = 5 per group) were analyzed. These samples constituted an independent histological validation cohort and were not necessarily derived from the same individuals included in the scRNA-seq analysis. Lung tissue sections embedded in paraffin underwent multiplex immunofluorescence staining utilizing a tyramide signal amplification (TSA) technique. Paraffin-embedded sections were processed by xylene-based dewaxing and graded rehydration, followed by antigen unmasking performed in EDTA solution (pH 9.0) under heating conditions. Endogenous peroxidase activity was inhibited with 3% hydrogen peroxide, and then serum blocking was performed with 3% BSA.

The following primary antibodies were used: anti-CD68 (ab289671; Abcam, Cambridge, UK),Anti-M-Ficolin (FCN1; ab112476; Abcam, Cambridge, UK),Anti-Osteopontin (SPP1; ab166709; Abcam, Cambridge, UK),Anti-COL1A2 (COL1A2; ab308455; Abcam, Cambridge, UK),Anti-SEMA3C (SEMA3C; PA5-103168; ThermoFisher, Waltham, MA, USA),Anti-neuropilin-1(NRP1; sc-5307; Santa Cruz, Dallas, TX, USA),Anti-PDGFR alpha (PDGFRA; ab203491; Abcam, Cambridge, UK),Anti-EBF1(EBF1; bs-12116R; Bioss, China),Anti-Apolipoprotein E (APOE; GB115764; Servicebio, China),Anti-CXCR4 (CXCR4; ab181020; Abcam, Cambridge, UK) were sequentially applied and incubated overnight at 4 °C. After washing, sections were incubated with HRP-conjugated secondary antibodies, followed by fluorophore-labeled tyramide (TSA) reagents (Alexa Fluor 488, Cy3, or Cy5). After each round of staining, bound antibodies were stripped by microwave heating in antigen retrieval buffer, while TSA signals remained covalently deposited at the target sites. Repeating the process three or more times enables triple-label staining, allowing simultaneous observation of three antigens on the same slide.

Lung tissue sections embedded in paraffin underwent three-color immunofluorescence staining with dual markers. In short, sections were deparaffinized and rehydrated using standard methods, then underwent heat-mediated antigen retrieval in citrate buffer with a pH of 6.0. After cooling to room temperature, slides were rinsed thoroughly with PBS. Tissue sections were permeabilized with 0.1% Triton X-100 for 10 min and subsequently blocked with 3% bovine serum albumin at room temperature for 30–60 min to reduce nonspecific binding. Primary antibodies from different host species, including anti-FCN1 (ab112476, Abcam, Cambridge, UK), anti-EBF1 (bs-12116R, Bioss, China), and anti-APOE (GB115764, Servicebio, China), were prepared according to the manufacturers’ instructions and incubated overnight at 4 °C.

After thorough PBS washes, the sections were incubated with species-matched secondary antibodies conjugated with Alexa Fluor 488 or Cy3 for one hour at room temperature in the dark. After completion of all staining steps, nuclei were counterstained with DAPI, and tissue autofluorescence was quenched. Slides were then mounted using an antifade mounting medium and imaged using an OLYMPUS FV3000 fluorescence microscope.

### 2.10 Immunohistochemical staining of human lung tissues

Human lung tissue sections were processed for immunohistochemical staining. Following deparaffinization in xylene and rehydration with graded ethanol, antigen retrieval was conducted in EDTA buffer (pH 9.0) by heating at 95–100 °C for 10–20 minutes. To quench endogenous peroxidase activity, samples were incubated with 3% hydrogen peroxide for 10 minutes at room temperature, then washed with PBS. A 5% bovine serum albumin solution was applied for 30–60 minutes at room temperature to block non-specific binding. The sections were incubated overnight at 4 °C with a primary antibody against NRP1 (sc-5307, Santa Cruz Biotechnology, Dallas, TX, USA). An HRP-linked secondary antibody was added and incubated at room temperature for 30 to 60 minutes. A DAB chromogenic substrate was used for signal development, and hematoxylin was used to counterstain the nuclei. In the end, sections were dehydrated using a series of ethanol concentrations, cleared with xylene, and mounted using neutral resin.

### 2.11 Cell thawing and culture

After removal from liquid nitrogen storage, THP-1 cells, MRC-5 cells, and sorted primary lung inflammatory monocyte/macrophage-enriched cells were rapidly transferred into a water bath and gently agitated for approximately 1 min to thaw. Once the cell suspension was completely thawed, it was transferred into a centrifuge tube containing 9 mL of pre-prepared culture medium: RPMI-1640 medium for THP-1 cells and primary human macrophages, and MEM medium for MRC-5 cells. The suspension was gently mixed and centrifuged at 1000 rpm for 5 min. After centrifugation, the supernatant was discarded, and the cell pellet was resuspended in 2 mL of complete medium. For THP-1 cells and primary human macrophages, the complete medium consisted of 10% FBS, 1% penicillin–streptomycin, and 89% RPMI-1640 medium. For MRC-5 cells, the complete medium consisted of 10% FBS, 1% penicillin–streptomycin, 1% non-essential amino acids (NEAA), and 88% MEM medium. The cell suspension was then mixed gently, transferred into a T25 culture flask, and supplemented with complete medium to a final volume of 5 mL.

### 2.12 Isolation of a CD45^+^CD11b^+^HLA-DR^+^CD64^+^CD14^+^CCR2^+^ inflammatory monocyte/macrophage-enriched population from RA-ILD lung tissue

Fresh peripheral lesional lung tissues from patients with RA-ILD were mechanically minced and enzymatically dissociated to generate single-cell suspensions. The digested tissues were sequentially filtered through 70-μm or 40-μm cell strainers to remove undigested fragments, followed by red blood cell lysis when necessary. Cells were washed with staining buffer containing PBS, 0.5–2% BSA or fetal bovine serum, and 2 mM EDTA. Cell number and viability were assessed before staining.

For flow cytometric staining, 1–2 × 10^6^ cells per tube were incubated with human Fc receptor blocking reagent for 10 min at 4 °C. Dead cells were excluded using a fixable viability dye, DAPI, or 7-AAD when required. Cells were then stained for 20–30 min at 4 °C in the dark with fluorochrome-conjugated antibodies against CD45, CD11b, HLA-DR, CD64, CD14, and CCR2/CD192. After staining, cells were washed twice and resuspended in sorting buffer containing 1–2% fetal bovine serum and 2 mM EDTA.

Cells were sorted on a BD FACSAria using a sequential gating strategy. Debris was first excluded according to FSC-A and SSC-A, followed by singlet selection using FSC-A versus FSC-H. CD45^+^ leukocytes were then gated, and CD45^+^CD11b^+^ myeloid cells were further selected. Within the CD45^+^CD11b^+^ population, HLA-DR^+^CD64^+^ cells were defined as macrophage/monocyte-lineage cells. CD14 and CCR2 expression was subsequently assessed, and CD45^+^CD11b^+^HLA-DR^+^CD64^+^CD14^+^CCR2^+^ cells were collected for downstream co-culture and functional assays. This surface-marker strategy enriches for recruited inflammatory monocyte-derived macrophage-like cells but does not define a transcriptionally pure FCN1^+^ population. FCN1 enrichment was assessed by immunofluorescence; because post-sort transcriptomic purity was not quantified, results from this fraction are described as arising from an inflammatory monocyte/macrophage-enriched population rather than exclusively from FCN1^+^ macrophages.

### 2.13 Lentiviral transduction of THP-1 cells (BeNa Culture Collection, BNCC358410)

THP-1 human monocytic cells (BeNa, BNCC358410) were maintained in RPMI-1640 supplemented with fetal bovine serum (10%) and penicillin–streptomycin (1%) under standard incubation conditions (37 °C, 5% CO_2_, humidified atmosphere). For lentiviral infection, cells were plated in antibiotic-free medium at a density of approximately 2–3 × 10^5^ cells/mL and allowed to equilibrate for 12–16 h before virus exposure. Recombinant lentiviruses encoding SEMA3C or the corresponding control constructs were applied at a multiplicity of infection of 50, together with polybrene (2 µg/mL; Beyotime, China, C0351). To improve infection efficiency, the cell suspension was subjected to centrifugation at 2500 rpm for 90 min. Following centrifugation, cells were returned to the incubator and cultured for an additional 12–16 h, after which the viral medium was removed and replaced with fresh complete medium. Transduction efficiency was subsequently assessed based on fluorescence signal intensity observed by fluorescence microscopy.

At 48 h after transduction, when cells exhibited stable proliferation, puromycin (2 µg/mL, Beyotime, China, ST551) was added for antibiotic selection. Selection was continued for 3–5 days until non-transduced control cells were completely eliminated and the transduction efficiency exceeded approximately 90%. Surviving cells were expanded and used for subsequent experiments. Where indicated, THP-1 cells were differentiated into macrophages by treatment with PMA (100 ng/mL, Beyotime, China, S1819) for 24 h, followed by stimulation with LPS (100 ng/mL, Sigma, USA, L2880) and IFN-γ (20 ng/mL, SinoBiological, China, 11725-HNAS) for 48 h prior to co-culture experiments.

### 2.14 Transwell co-culture system

A Transwell co-culture system was established using MRC-5 fibroblasts and macrophages derived either from patients with RA-ILD or from THP-1 cells. First, recovered fibroblasts were seeded into the lower chamber and cultured under the fibroblast growth conditions described above to allow cell recovery and stable adhesion. Subsequently, macrophages were seeded into permeable membrane inserts positioned above the fibroblast layer. The Transwell membrane had a pore size of 0.4 μm, which allowed the diffusion of soluble factors while preventing cell migration across the membrane, thereby enabling paracrine interactions without direct cell–cell contact. Parallel monoculture groups, lentivirally transduced macrophage groups, and macrophage groups directly isolated from patient lung tissues were included to distinguish cell type–specific effects from cell–cell interaction effects.

During co-culture with THP-1-derived macrophages, interleukin-1β (IL-1β, 2 ng/mL; Servicebio, China, GB300002-H) was added to the culture system to mimic an inflammatory microenvironment. In some experimental groups, an NRP1 receptor antagonist (10 μM; MedChemExpress, USA, HY-10799) was added simultaneously to inhibit NRP1 signaling activity. For exogenous stimulation experiments, recombinant human SEMA3C protein (20 ng/mL; MedChemExpress, USA, HY-P71282A) or recombinant human TGF-β protein (5 ng/mL; MedChemExpress, USA, HY-P78213) was directly added to the fibroblast culture.

### 2.15 CCK-8 assay

Cell viability was assessed using the Cell Counting Kit-8 assay (CCK-8; Beyotime, China, C0042). THP-1 monocytes were transduced with lentivirus at different multiplicities of infection (MOI = 0, 10, 20, 50, and 100), with the MOI = 0 group serving as the negative control (NC). After transduction, cells were cultured in the presence of different concentrations of interleukin-1β (IL-1β; 0, 2, 4, 6, and 8 ng/mL; Servicebio, China) for 48 h. Subsequently, 10 μL of CCK-8 reagent was added to each well and incubated at 37 °C for 1.5 h. Absorbance was then measured at 450 nm to evaluate the effects of different MOI conditions and inflammatory stimulation on THP-1 cell viability. Fibroblasts were seeded in 96-well plates and then co-cultured with macrophages in the presence of inflammatory stimulation using interleukin-1β (IL-1β, 2 ng/mL; Servicebio, China). After 36 h of co-culture, CCK-8 reagent (10 μL per well) was applied and allowed to incubate at 37 °C for 1.5 hours.

### 2.16 ELISA assay

The concentrations of SEMA3C in the culture supernatants were quantified using a human Sema Domain–containing immunoglobulin Ig3C (SEMA3C) ELISA kit (Jianglai Bio, China, JL30524-96T) following the manufacturer’s instructions. After 48 h of macrophage–fibroblast co-culture, cell culture supernatants were collected and centrifuged at 3000 rpm for 10 min to remove debris. The supernatants were then incubated in pre-coated ELISA plates with specific capture antibodies, followed by detection antibodies and HRP-conjugated secondary antibodies. Color development was performed using TMB substrate, and absorbance was measured at 450 nm.

### 2.17 Immunofluorescence staining of cultured fibroblast and macrophage

Cultured fibroblasts grown on glass coverslips were processed for immunofluorescence analysis. Cells were fixed in 4% paraformaldehyde for 15 min, followed by permeabilization with 0.3% Triton X-100 for 10 min. Non-specific binding was reduced by incubation with 3% bovine serum albumin at room temperature for 30 min. The cells were then incubated overnight at 4 °C with primary antibodies targeting EBF1, PDGFRA, CXCR4 or APOE. After washing, appropriate Alexa Fluor 488-, Cy3-, or Cy5-labeled secondary antibodies were applied for 1 h at room temperature in the dark. Nuclear staining was performed using DAPI (5 μg/mL) for 10 min. Coverslips were mounted in antifade mounting medium, and images were obtained using a Nikon Eclipse C1 fluorescence microscope.

### 2.18 Statistical analysis of scRNA-seq data

Statistical analyses of the scRNA-seq data were primarily performed at the donor level, with the three study groups comprising 3 RA-ILD, 4 HC, and 5 IPF independent donors. Gene-expression counts were aggregated within each donor and cell type to generate pseudobulk profiles. Because of the limited number of donors, nonparametric statistical tests were used without assuming normality. Comparisons between two independent groups were performed using the two-sided Mann–Whitney U test, whereas comparisons among the three groups were performed using the Kruskal–Wallis test followed by Dunn’s multiple-comparison test. Differences in cell-type proportions were evaluated using the same donor-level approach. P values obtained from genome-wide differential expression analyses were adjusted for multiple testing using the Benjamini–Hochberg method, and genes with an FDR < 0.05 were considered statistically significant. Functional scores, including inflammation and tissue repair scores, were calculated at the individual-cell level using UCell and visualized using violin and box plots to illustrate their distributions across cell populations and study groups. Individual cells were not considered independent biological replicates for donor-level statistical inference.

Gene Ontology enrichment analysis was performed using Metascape with the whole genome as the background and a cumulative hypergeometric test. GO-term P values were corrected by the Benjamini–Hochberg method, and terms with FDR (q) < 0.05 were retained.

### 2.19 Statistical analysis of *in vitro* experiments

Cell-based experiments were analyzed using GraphPad Prism 9 (GraphPad Software). Data are summarized as mean ± standard deviation (SD) from three biologically independent experiments; technical wells or fields were averaged within each experiment and were not treated as independent n values. Because n = 3 does not permit a reliable assessment of normality, the P values from the prespecified one-way ANOVA and *post hoc* comparisons are regarded as exploratory. Accordingly, interpretation emphasizes the direction and reproducibility of marker changes across independent experiments rather than formal proof based on distributional assumptions. All tests were two-sided, and P < 0.05 was used as an exploratory threshold.

## 3 Results

### 3.1 Single-cell sequencing reveals a cellular landscape enriched in inflammatory features in the studied RA-ILD cohort

To investigate the mechanisms driving pulmonary fibrosis in RA-ILD, human lung tissues were prepared as single-cell suspensions and subjected to single-cell RNA sequencing, and fresh lung tissue samples from healthy controls (HCs), RA-ILD patients, and IPF patients were analyzed (**Figure 1a**). Detailed patient information is provided in **Figure 1b** and **Supplementary Table 1**. Histopathologically, all RA-ILD patients showed a fibrotic NSIP pattern, although the HRCT findings included mixed UIP/NSIP or indeterminate-for-UIP/NSIP features; in contrast, all IPF samples showed a UIP pattern on both HRCT and histopathological evaluation.

To validate the biological relevance of the sequenced samples, lung tissues from the three groups were simultaneously subjected to H&E staining. The results showed that the HCs exhibited a normal alveolar architecture, the RA-ILD group displayed macrophage accumulation and aggregation, alveolar hemorrhage and edema, and fibroblast aggregation, while the IPF group showed fibroblast foci, epithelial metaplasia, and aggregation of some immune cells, consistent with the characteristic features of each disease (**Figure 1c**).

After quality control and batch-effect correction, the single-cell sequencing data were integrated into a dataset containing 71,626 cells (**Figures 1d, e**). Following dimensionality reduction and clustering, all cell populations were annotated (**Figure 1f**; **Supplementary Figure S1a**). We found that the proportion of fibroblasts did not show a marked change among the three groups, whereas the proportion of macrophages, compared with the HCs (12.8%), was increased in the RA-ILD group (17%) and was markedly increased in the IPF group (25.3%) (**Figure 1g**). In addition, group-wise scoring revealed that inflammation-related scores were higher in RA-ILD than in the other two groups (**Figure 1h**).

Given the prominent involvement of myeloid cells in RA-ILD, we further examined neutrophils, monocytes, and macrophage subpopulations (**Supplementary Figure S1b**). Their identities were supported by canonical and discriminatory marker expression (**Supplementary Figure S1c**) and further validated by lineage-specific module-score analyses, which showed preferential enrichment of neutrophil, monocyte, and macrophage programs in their respective annotated populations (**Supplementary Figures S1d–i**). In addition, coordinated detection of neutrophil-core markers was not observed in macrophages, and doublet-score analysis showed no evidence of widespread doublet contamination, further supporting the robustness of the myeloid-cell annotations.

### 3.2 FCN1^+^ inflammatory macrophages are enriched in the studied RA-ILD lungs

Given the elevated inflammatory signature in RA-ILD and the increase in macrophage proportions, we next characterized macrophage heterogeneity across disease conditions. We classified macrophages into FABP4^+^, SPP1^+^, FCN1^+^, FOLR2^+^, and proliferative subpopulations. FABP4^+^ macrophages accounted for the highest proportion in HC lung tissues (67.3%), whereas the IPF group was dominated by SPP1^+^ macrophages (65.2%). In the three RA-ILD donors analyzed, FCN1^+^ macrophages accounted for 36% of the macrophage compartment and co-expressed macrophage- and monocyte-associated markers (**Figures 2a–d**; **Supplementary Figure S2a**). FCN1^+^ macrophages also showed the highest inflammation scores (**Figure 2e**). These data demonstrate enrichment relative to the IPF and HC groups in this cohort; they do not establish specificity to RA-ILD across inflammatory lung diseases.

**Figure 2:**
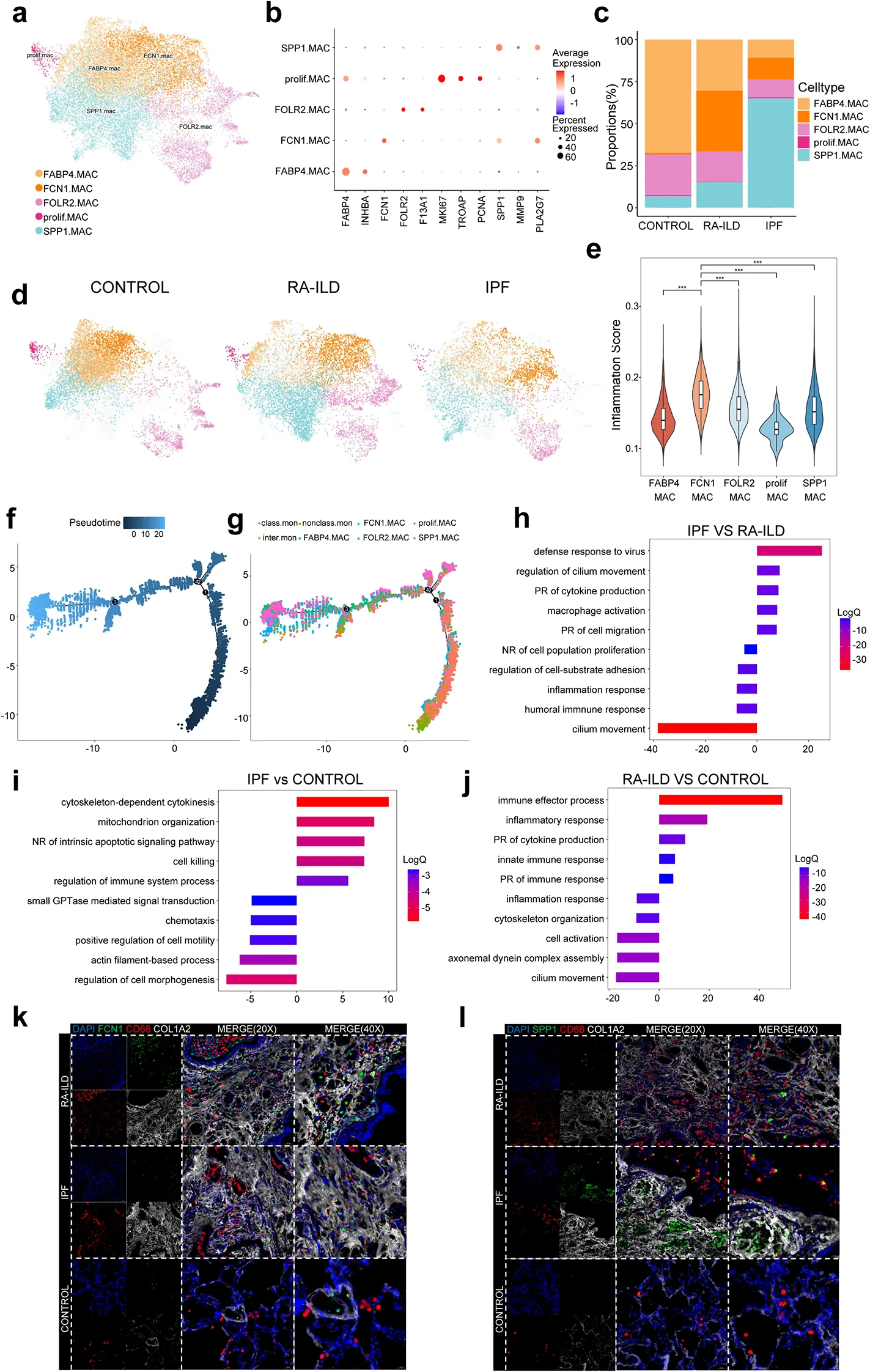
Characterization and functional analysis of macrophage subpopulations in RA-ILD and IPF lung tissues. **(a)** UMAP visualization of all macrophage populations derived from human lung single-cell RNA sequencing datasets. **(b)** Bubble plot showing the expression of representative marker genes across macrophage subsets, including FABP4^+^, FCN1^+^, SPP1^+^, FOLR2^+^ and proliferative macrophages. **(c)** Stacked bar plot depicting the relative proportions of each macrophage subset in HCs, RA-ILD, and IPF samples. **(d)** UMAP plots showing macrophage distributions across the three groups (HCs, RA-ILD, and IPF). **(e)** Violin plot comparing inflammation scores among macrophage subsets in different disease groups. **(f, g)** Pseudotime trajectory analysis of macrophages using Monocle2, visualized by pseudotime **(f)** and cell subtype identity **(g)**. **(h–j)** GO enrichment analysis of differentially expressed genes in FCN1^+^ macrophages comparing RA-ILD vs. IPF **(h)**, IPF vs. RA-ILD **(i)**, and IPF vs. CONTROL **(j)**. RA-ILD vs. CONTROL GO terms were tested by cumulative hypergeometric tests and corrected by the Benjamini–Hochberg method; FDR **(q)** < 0.05 was considered significant. **(k, l)** Representative immunofluorescence staining images showing the localization of FCN1+ macrophages **(k)** and SPP1+ macrophages **(l)** in relation to collagen deposition in RA-ILD and IPF lung tissues. Nuclei were counterstained with DAPI (blue).

Using Monocle to infer cell developmental trajectories along pseudotime (**Figures 2f, g**), we found that macrophage differentiation along pseudotime progressed from classical and non-classical monocytes toward macrophages, including both SPP1^+^ and FCN1^+^ macrophages. These results suggest a monocyte-to-macrophage continuum in both RA-ILD and IPF, with FCN1^+^ macrophages occupying an inflammatory macrophage state related to, but distinct from, circulating monocytes. Gene Ontology enrichment analysis of differentially expressed genes among the three cohorts (**Figures 2h-j**) showed that macrophages in RA-ILD exhibited enrichment in processes such as immune response, cell migration, and cell-substrate adhesion, suggesting that macrophages may be actively recruited to lesion sites and participate in inflammation or tissue remodeling. In contrast, the HC group exhibited enrichment in pathways related to cell activation and immune responses. Differential gene GO enrichment between the IPF and RA-ILD groups indicated that macrophages in the RA-ILD group participate in inflammatory responses, cell–matrix adhesion processes, and disease progression through macrophage reactivation mechanisms. The GO enrichment analysis of differentially expressed genes in FCN1+ macrophages revealed similar findings, showing that FCN1+ macrophages in the RA-ILD group are involved in a broader range of inflammatory processes (**Supplementary Figure S2b, c**).

To investigate the spatial distribution of FCN1+ and SPP1+ macrophages, immunofluorescence staining was performed on paraffin-embedded lung tissue sections from the normal, RA-ILD, and IPF groups (**Figures 2k, l**). The results showed that FCN1 expression was mainly observed in the RA-ILD group and closely co-localized with the macrophage marker CD68 within collagen-rich areas. In contrast, SPP1 expression was markedly increased in the IPF group, with partial co-localization with the macrophage marker CD68, while another portion was secreted into the collagenous tissue. In addition, a small amount of SPP1 expression was also observed in the RA-ILD group, mainly localized within the lumen. Quantitative analysis of the immunofluorescence staining further confirmed increased FCN1-positive staining in RA-ILD and increased SPP1-positive staining in IPF, consistent with the representative images (**Supplementary Figures S2d, e**).

### 3.3 Matrix fibroblasts are linked to a CXCR4^+^ inflammation-associated lipofibroblast state in RA-ILD

We next characterized fibroblast heterogeneity, identifying three main subpopulations: alveolar fibroblasts (NPNT^+^, CES1^+^), matrix fibroblasts (EBF1^+^, TSHZ2^+^), and myofibroblasts (POSTN^+^, CTHRC1^+^) (**Figures 3a, b**; **Supplementary Figure S3a**). Lipofibroblasts were identified by APOE expression; among these, a subset co-expressing CXCR4 was designated as inflammation-associated lipofibroblasts. Non-activated lipofibroblasts were mainly observed in HCs, inflammation-associated lipofibroblasts constituted the largest fibroblast subset in the RA-ILD samples, and myofibroblasts predominated in IPF (**Figures 3c, d**). Although the overall fibroblast proportion did not differ significantly among groups, their subtype distributions differed, suggesting disease-associated variation in fibroblast states.

**Figure 3:**
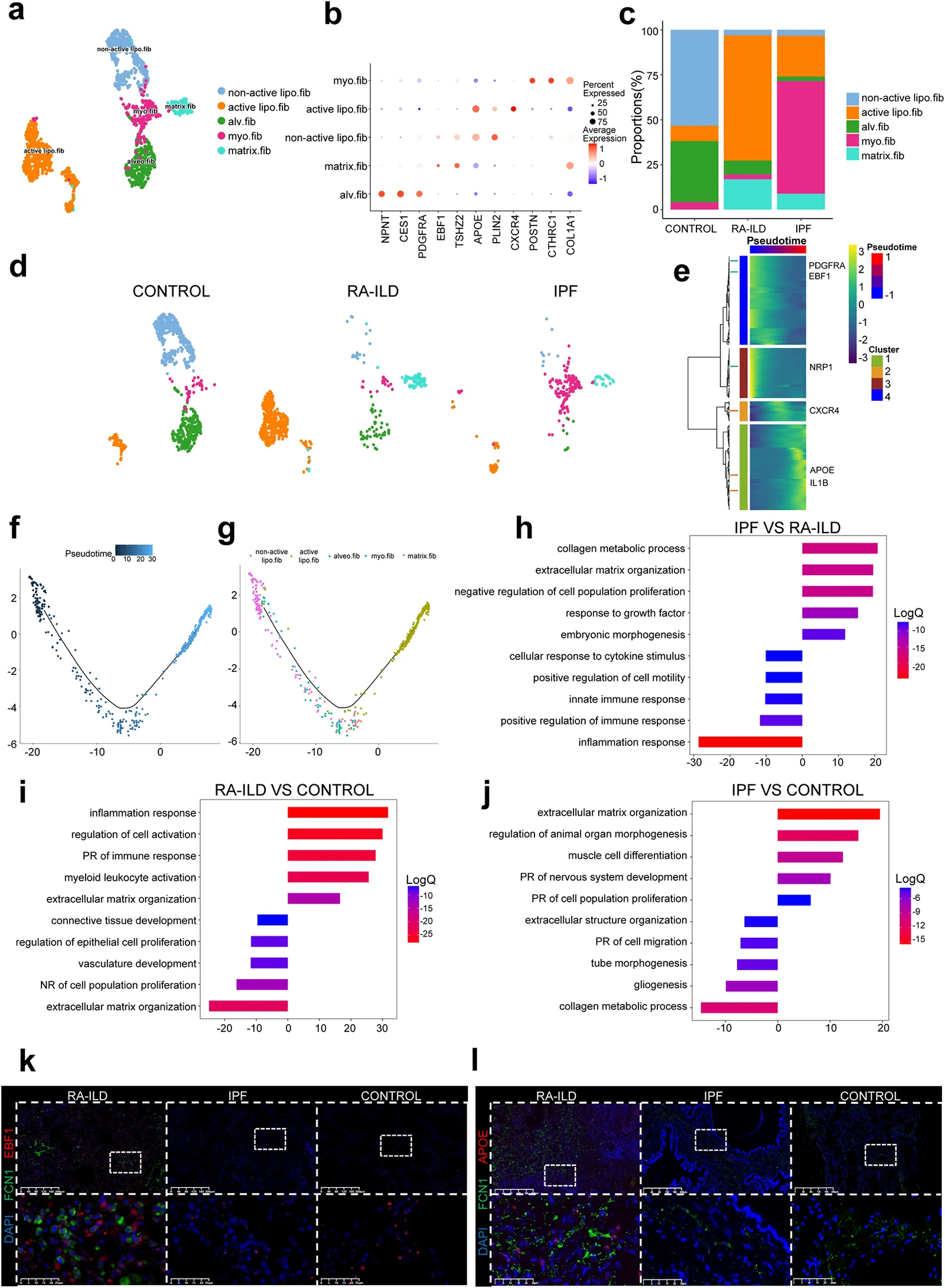
Characterization and trajectory analysis of fibroblast subpopulations in RA-ILD and IPF lung tissues. **(a)** UMAP visualization of all fibroblast populations derived from human lung single-cell RNA sequencing (scRNA-seq) datasets. **(b)** Bubble plot showing representative marker genes defining fibroblast subsets, including alveolar fibroblasts, matrix fibroblasts, myofibroblasts, non-activated APOE^+^ lipofibroblast-like fibroblasts, and CXCR4^+^ inflammation-associated APOE^+^ fibroblasts. **(c)** Stacked bar plot depicting the relative proportions of fibroblast subsets in HCs, RA-ILD, and IPF groups. **(d)** UMAP plots displaying fibroblast distributions among the three groups (HCs, RA-ILD, and IPF). **(e)** Heatmap illustrating the dynamic expression of fibroblast subset-specific genes along the pseudotime trajectory. **(f, g)** Pseudotime trajectory analysis of fibroblasts reconstructed using Monocle2, visualized by pseudotime **(f)** and fibroblast subtype identity **(g)**. **(h–j)** GO enrichment analysis of differentially expressed genes in fibroblasts comparing RA-ILD vs. IPF **(h)**, IPF vs. RA-ILD **(i)**, and RA-ILD vs. CONTROL **(j)**. IPF vs. CONTROL GO-term P values were corrected by the Benjamini–Hochberg method, with FDR (q) < 0.05 considered significant. **(k, l)** Representative immunofluorescence staining images showing the colocalization of matrix fibroblast markers EBF1 **(k)** or APOE **(l)** with FCN1+ macrophages in RA-ILD, IPF, and HCs lung tissues. Nuclei were counterstained with DAPI (blue).

Differential gene expression analysis revealed that CXCR4 and EREG were highly expressed only in activated lipofibroblasts (**Figure 3e**, **Supplementary Figure S3b**). CXCR4 and EREG are genes associated with cell migration, inflammation, and tissue repair and regeneration; however, these genes were not highly expressed in non-activated lipofibroblasts. In addition, trajectory analysis in the RA-ILD group showed that matrix fibroblasts differentiated into CXCR4+ activated lipofibroblasts over time (**Figures 3f, g**). However, in the IPF group, we identified the presence of a subset of lipofibroblasts; however, these cells in the IPF group did not express CXCR4 or EREG (**Supplementary Figure S3b**). Functional enrichment analysis of fibroblast differentially expressed genes indicated that RA-ILD exerts its effects by promoting an inflammatory microenvironment with immune activation, which may drive fibroblasts toward inflammation-associated phenotypes (**Figures 3h–j**). Similarly, GO enrichment analysis of differentially expressed genes in lipofibroblasts revealed that, compared with other groups, lipofibroblasts in the RA-ILD group are more involved in inflammatory processes (**Supplementary Figures S3d, e**). Moreover, CXCR4-expressing lipofibroblasts exhibited significantly elevated inflammation and tissue repair scores. In IPF, myofibroblasts were involved in collagen metabolism and structural remodeling, which explains the increased collagen deposition observed in lung tissues of IPF patients (**Supplementary Figure S3c**).

Immunofluorescence analysis showed EBF1-expressing fibroblasts in spatial proximity to FCN1-expressing cells in the RA-ILD sections examined (**Figure 3k**), and APOE staining showed a similar spatial relationship (**Figure 3l**). To further validate the APOE^+^CXCR4^+^ fibroblast population identified by single-cell analysis, we performed co-immunofluorescence staining for APOE, CXCR4, and the fibroblast marker PDGFRA. Prominent co-localization of APOE and CXCR4 with PDGFRA was observed in RA-ILD lung tissues, whereas APOE–PDGFRA co-localization was only occasionally detected in IPF and control tissues (**Supplementary Figure S3f**). Quantitative analysis of the immunofluorescence signals further supported these differences among the three groups (**Supplementary Figures S3g–i**). Together with the single-cell data, these findings support the presence of an APOE^+^CXCR4^+^ fibroblast population and its association with FCN1^+^ inflammatory macrophages in this RA-ILD cohort. Spatial proximity does not by itself establish direct signaling, and the absence of other CTD-ILD comparators precludes a claim of RA-ILD specificity.

### 3.4 An RA-ILD-enriched SEMA3C–NRP1 signaling pattern links inflammatory macrophage populations to matrix fibroblasts

CellChat analysis indicated greater predicted intercellular communication in RA-ILD than in IPF in terms of interaction number and strength (**Figure 4a**). SEMA3 signaling ranked highly in the RA-ILD group (**Figure 4b**). When FCN1^+^ macrophages were specified as sender cells and fibroblast subsets as receivers, IPF interactions were dominated by SPP1-related pairs, whereas the RA-ILD samples showed SEMA3C-related pairs involving matrix fibroblasts (**Figures 4c–e**). Analysis using all macrophage populations as senders produced a concordant pattern: the SEMA3C–NRP1 pair was detected in the RA-ILD group but not in the IPF or HC groups in this dataset (**Supplementary Figures S4a–d**). This is a within-cohort comparison and should not be interpreted as disease specificity across CTD-ILDs. SEMA3C transcripts were observed in both FCN1^+^ and SPP1^+^ macrophage subsets in RA-ILD, whereas NRP1 was expressed across fibroblast subsets (**Figures 4f, g**). Thus, the transcriptomic data support a macrophage-to-fibroblast signaling hypothesis but do not establish FCN1^+^ macrophages as the exclusive cellular source of SEMA3C.

**Figure 4:**
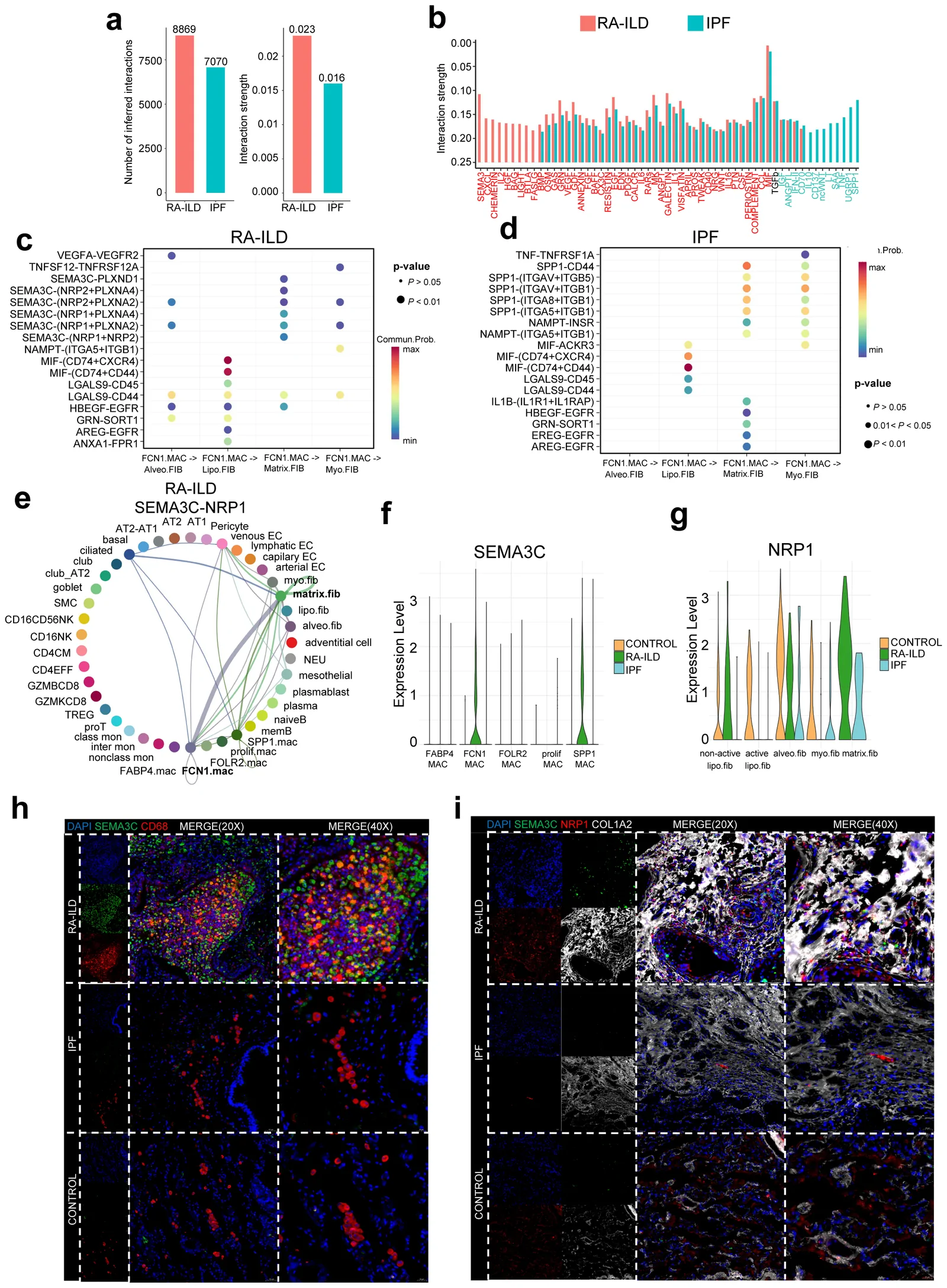
Macrophage–fibroblast crosstalk analysis reveals the activation of the SEMA3C–NRP1 axis in RA-ILD. **(a)** Bar plots showing the overall number and strength of predicted cell–cell interactions between RA-ILD and IPF samples. **(b)** Comparison of interaction strength across major signaling pathways between RA-ILD and IPF groups. **(c, d)** Bubble plots displaying the top ligand–receptor signaling pathways between FCN1+ macrophages (as ligand sources) and fibroblast subsets (as targets) in RA-ILD **(c)** and IPF **(d)** lungs. **(e)** Chord diagram illustrating the SEMA3C–NRP1 ligand–receptor interaction network between macrophages and fibroblasts in RA-ILD lungs. **(f)** Violin plot showing the expression levels of SEMA3C across macrophage subsets in HCs, RA-ILD, and IPF groups. **(g)** Violin plot showing NRP1 expression in fibroblast subsets under the same conditions. **(h)** Representative immunofluorescence staining of macrophage marker CD68 (green) and ligand SEMA3C (red) in RA-ILD, IPF, and HCs lung tissues, demonstrating co-localization in fibrotic lesions. Nuclei were counterstained with DAPI (blue). **(i)** Representative immunofluorescence staining showing the co-localization of receptor NRP1 (green) with SEMA3C (red) and collagen I (white) in fibrotic regions of RA-ILD and IPF lungs. High-magnification insets highlight macrophage–fibroblast interfaces.

IHC showed higher NRP1 signal in RA-ILD than in IPF lung sections (**Supplementary Figure S4e**). Multiplex immunofluorescence showed SEMA3C signal co-localized with CD68 and FCN1 and located near NRP1 within collagen-rich regions (**Figures 4h, i**; **Supplementary Figure S4h**). Quantitative analysis further showed increased SEMA3C and CD68 signals, together with increased COL1A2-, NRP1-, and SEMA3C-positive fluorescence areas in RA-ILD lung tissues compared with controls (**Supplementary Figures S4f, g, i**). These findings support macrophage-associated SEMA3C expression and its spatial apposition to NRP1^+^ stromal cells, although they do not establish an exclusive cellular source of SEMA3C or direct SEMA3C–NRP1 signaling.

### 3.5 An RA-ILD inflammatory monocyte/macrophage-enriched population is associated with APOE^+^CXCR4^+^ fibroblast-state changes

To functionally examine the predicted axis, we isolated a CD45^+^CD11b^+^HLA-DR^+^CD64^+^CD14^+^CCR2^+^ inflammatory monocyte/macrophage-enriched population from RA-ILD and HC lung tissues (**Figure 5a**; **Supplementary Figure S5a**). Immunofluorescence showed higher FCN1 signal in the RA-ILD-derived fraction (**Figure 5b**), and ELISA showed higher SEMA3C concentrations in its culture supernatant (**Figure 5c**). Because FCN1 purity was not quantified after sorting, these findings cannot assign SEMA3C secretion exclusively to FCN1^+^ macrophages. In Transwell co-culture with MRC-5 fibroblasts, the RA-ILD-derived fraction was associated with lower EBF1/PDGFRA and higher APOE/CXCR4 signals than the HC-derived fraction (**Figures 5d–k**). These results indicate a marker-defined fibroblast-state shift associated with soluble factors from the enriched inflammatory myeloid population; they do not, by themselves, demonstrate acquisition of a complete profibrotic functional phenotype.

**Figure 5:**
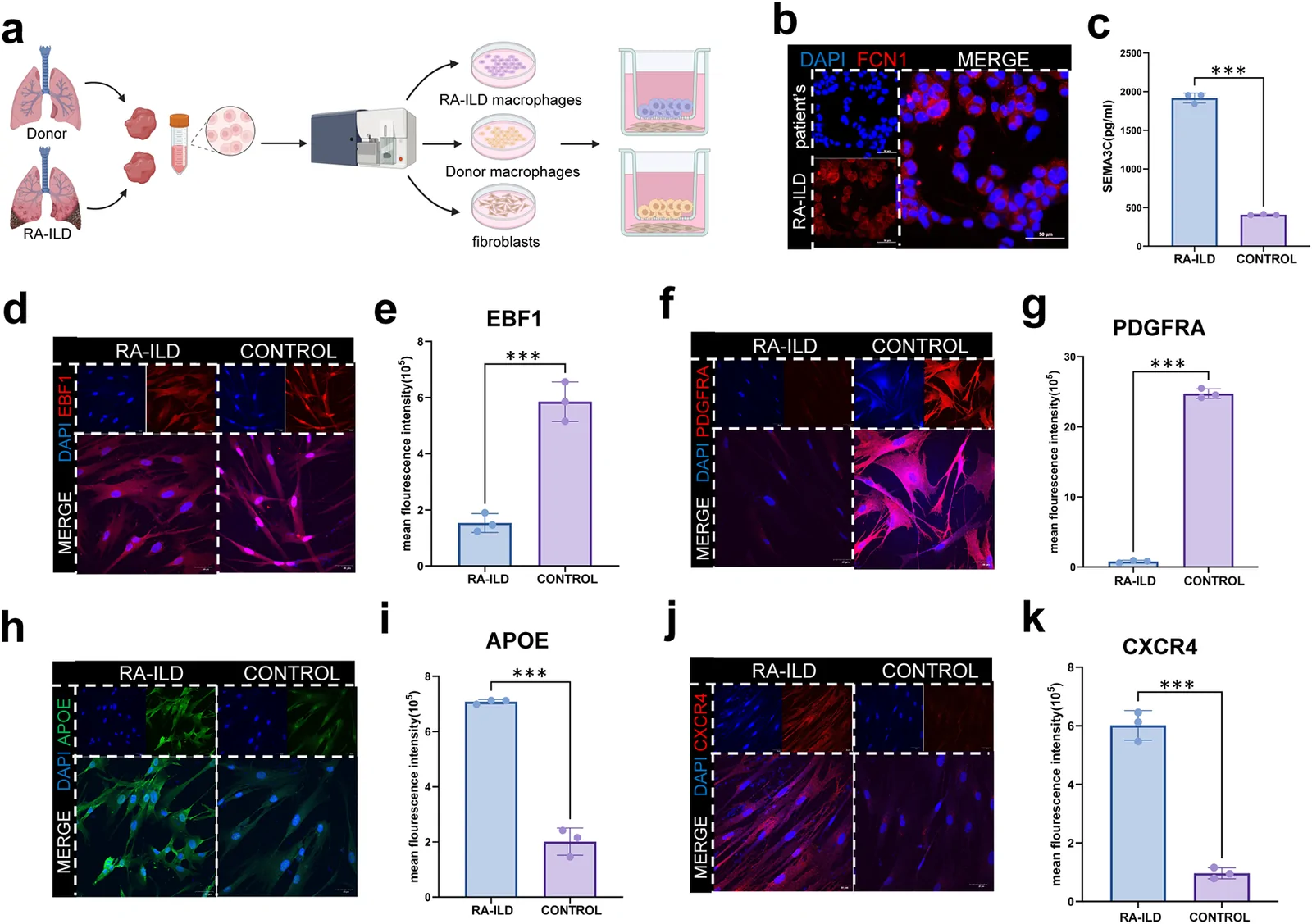
*In vitro* validation of macrophage–fibroblast crosstalk mediated by SEMA3C signaling in RA-ILD. **(a)** Schematic of the Transwell co-culture system using an inflammatory monocyte/macrophage-enriched fraction derived from RA-ILD or HC lung tissue and MRC-5 human fetal lung fibroblast cells. The sorted fraction was enriched for FCN1 signal but was not established to be transcriptionally pure FCN1^+^ macrophages. **(b)** Immunofluorescence showing FCN1 expression in RA-ILD-derived inflammatory monocyte/macrophage-enriched cells. **(c)** ELISA measurement of SEMA3C in culture supernatants from the sorted inflammatory myeloid fractions. Results are from three biologically independent cell preparations; technical measurements were averaged within each preparation. P values are exploratory. **(d–k)** Representative immunofluorescence and quantitative analysis of MRC-5 fibroblast-state markers after co-culture with the sorted inflammatory myeloid fractions. **(d, e)** EBF1 signal was lower in MRC-5 cells under the RA-ILD-derived co-culture condition than under the HC-derived condition. **(f, g)** PDGFRA signal was lower in MRC-5 cells exposed to the RA-ILD-derived fraction. **(h, i)** APOE signal was higher in MRC-5 cells co-cultured with the RA-ILD-derived fraction. **(j, k)** CXCR4 signal was also higher under the RA-ILD-derived co-culture condition. Nuclei were counterstained with DAPI (blue). Quantification is shown as mean ± SD from n = 3 biologically independent experiments; technical fields were averaged within each experiment. P values are exploratory (*P < 0.05, **P < 0.01, ***P < 0.001).

### 3.6 Exogenous SEMA3C induces a marker-defined fibroblast-state shift

To examine whether SEMA3C could reproduce this marker pattern, MRC-5 human fetal lung fibroblast cells—not adult primary lung fibroblasts—were treated with recombinant SEMA3C (20 ng/mL) (**Figure 6a**). Immunofluorescence staining confirmed NRP1 expression in MRC-5 cells, with no marked change in NRP1 signal following SEMA3C treatment, as supported by quantitative analysis (**Supplementary Figure S5b**). SEMA3C exposure reduced EBF1 and PDGFRA signals and increased APOE and CXCR4 signals (**Figures 6b–j**). These data show that SEMA3C is sufficient to induce the measured marker-defined state change in MRC-5 cells. Because collagen synthesis, α-SMA, matrix contraction, ECM secretion, migration, and proliferation were not evaluated, we do not equate this state change with a fully established profibrotic phenotype.

**Figure 6:**
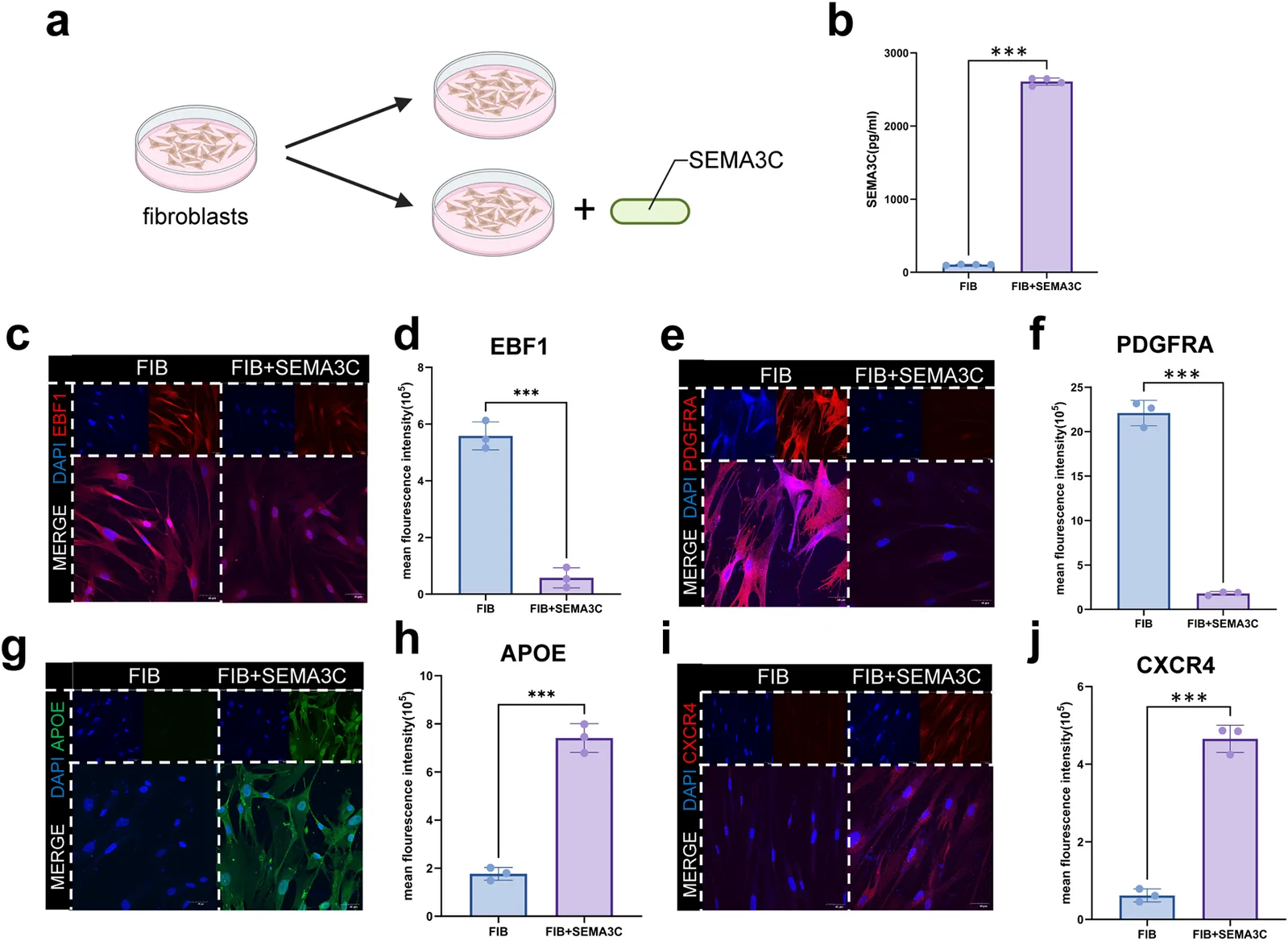
Exogenous SEMA3C induces a marker-defined state change in MRC-5 fibroblasts. **(a)** Experimental setup in which MRC-5 human fetal lung fibroblast cells were treated with recombinant SEMA3C or vehicle control. MRC-5 cells are a cell line and are not referred to as primary fibroblasts. **(b)** ELISA quantification confirming high SEMA3C levels in the treatment group. **(c–j)** Representative immunofluorescence staining and quantitative analysis of fibroblast marker expression following SEMA3C stimulation. **(c, d)** Expression of EBF1 (red) was markedly reduced in fibroblasts treated with SEMA3C compared with vehicle-treated controls. **(e, f)** PDGFRA signal was lower following SEMA3C exposure. **(g, h)** APOE signal was higher in SEMA3C-treated MRC-5 cells. **(i, j)** CXCR4 signal was also higher in the SEMA3C-treated group. Nuclei were counterstained with DAPI (blue). Quantification is shown as mean ± SD from n = 3 biologically independent experiments; technical fields were averaged within each experiment. P values are exploratory (*P < 0.05, **P < 0.01, ***P < 0.001). These markers indicate a state change but do not establish a complete profibrotic functional phenotype.

### 3.7 NRP1 inhibition attenuates SEMA3C-associated fibroblast marker reprogramming

To examine whether this effect was receptor-dependent, we engineered THP-1-derived macrophages to overexpress SEMA3C (**Figures 7a–d**). These cells produced higher levels of extracellular SEMA3C (**Figure 7e**). In the Transwell co-culture system, MRC-5 fibroblasts exhibited decreased EBF1 and PDGFRA signals and increased APOE and CXCR4 signals. Treatment with an NRP1 antagonist attenuated these fibroblast marker changes (**Figures 7f–m**). Taken together, these findings support the involvement of NRP1 in the fibroblast marker response associated with macrophage-derived SEMA3C.

**Figure 7:**
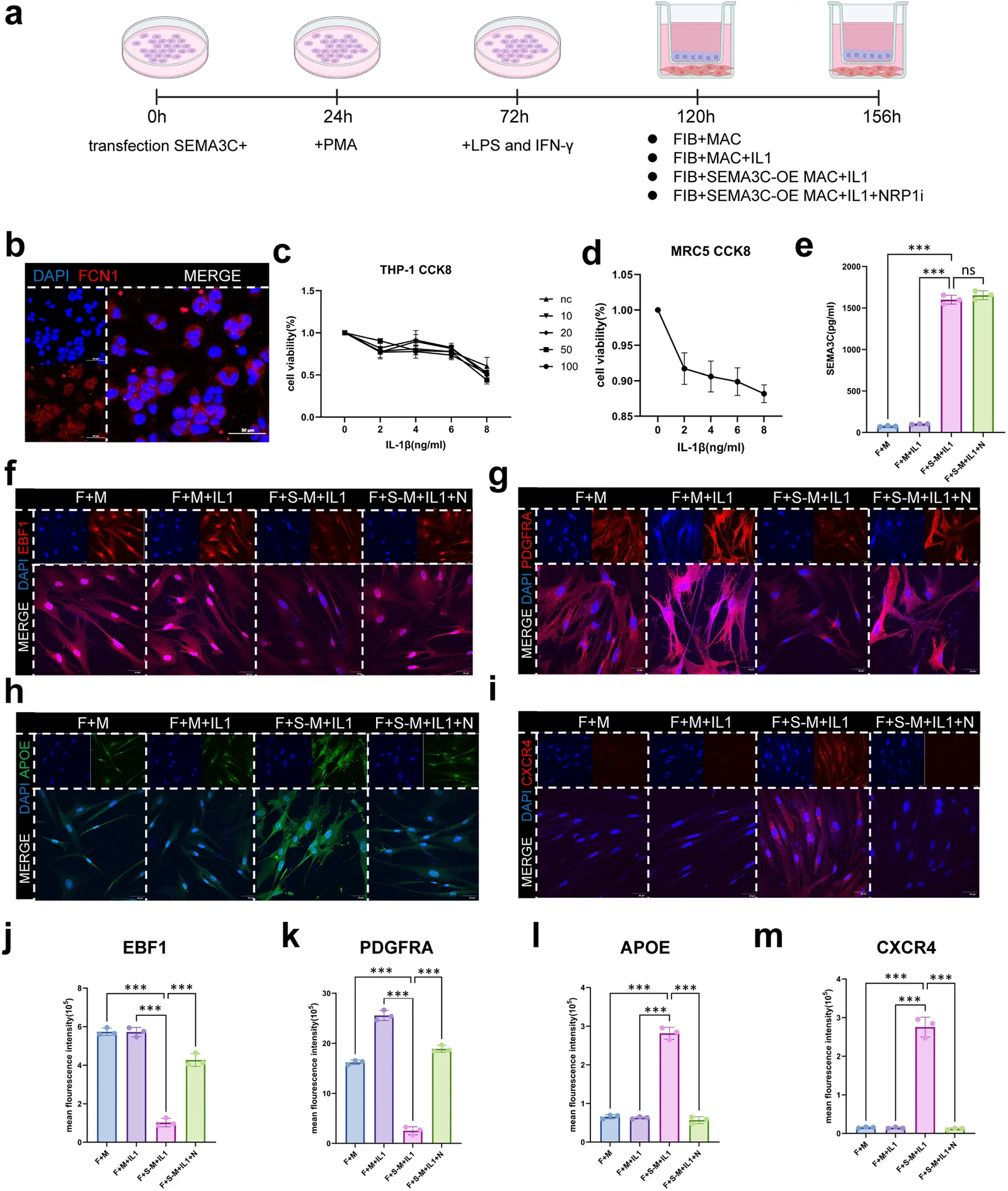
Macrophage-derived SEMA3C induces NRP1-dependent fibroblast marker reprogramming. **(a)** Experimental workflow showing the transfection and co-culture design. THP-1 monocytes were transduced with lentiviral vectors encoding SEMA3C, differentiated into macrophages using PMA, and activated by LPS and IFN-γ. Afterward, macrophages were co-cultured with MRC-5 fibroblasts in Transwell chambers under four conditions: FIB+MAC, FIB+MAC+IL1β, FIB+SEMA3C-OE MAC+IL1β, and FIB+SEMA3C-OE MAC+IL1β+NRP1 receptor antagonist. **(b)** Immunofluorescence staining confirming FCN1 expression in SEMA3C-transfected macrophages. (c–d) CCK-8 assays assessing the effect of IL-1β concentration on THP-1 macrophage viability **(c)** and MRC-5 fibroblast viability **(d)**. **(e)** ELISA measurement of extracellular SEMA3C. (f–i) Representative immunofluorescence of MRC-5 fibroblast markers under the indicated co-culture conditions. **(f, g)** EBF1 and PDGFRA signals were lower in fibroblasts co-cultured with SEMA3C-overexpressing macrophages. **(h, i)** APOE and CXCR4 signals were higher under the same condition, whereas NRP1 inhibition attenuated these marker changes. **(j–m)** Quantitative fluorescence analysis of EBF1, PDGFRA, APOE, and CXCR4. Data are mean ± SD from n = 3 biologically independent experiments; technical fields were averaged within each experiment. P values are exploratory (*P < 0.05, **P < 0.01, ***P < 0.001).

## 4 Discussion

SEMA3C signals through receptor complexes containing neuropilins, plexins, and additional co-receptors, thereby regulating cell migration, attraction, repulsion, and intercellular communication (18, 19). Previous studies have shown that high expression of SEMA3C is positively correlated with the severity of liver fibrosis in chronic hepatitis, and that upregulation of SEMA3C and NRP2 can enhance TGF-β–mediated myofibroblast activation, thereby aggravating fibrotic progression (13). In addition, the binding of SEMA3C to NRP2 can activate MAPK signaling pathways and promote liver metastasis of colorectal cancer (20). In recent years, SEMA3C has also been reported to be involved in the development and progression of various diseases, including glioblastoma, hepatocellular carcinoma, pancreatic cancer, thyroid cancer, and cervical cancer, with mechanisms closely related to regulation of the immune microenvironment (21–25).

Within the present comparison of three RA-ILD donors, four HCs, and five IPF donors, FCN1^+^ macrophages were enriched in RA-ILD, SPP1^+^ macrophages predominated in IPF, and FABP4^+^ macrophages predominated in HCs. FCN1^+^ inflammatory macrophages have also been described in COVID-19, sarcoidosis, and other inflammatory lung conditions; therefore, their presence is not intrinsically specific to RA-ILD. Our data instead suggest that the combination of FCN1^+^ macrophage enrichment, macrophage-associated SEMA3C, and an NRP1^+^ fibroblast response characterizes the RA-ILD samples studied here. Testing whether this combination distinguishes RA-ILD from other CTD-ILDs will require external cohorts including systemic-sclerosis-, Sjögren’s-syndrome-, myositis-, and SLE-associated ILD. One technical consideration is that FCN1^+^ macrophages were much less abundant in IPF than in RA-ILD. Therefore, CellChat comparisons specifying FCN1^+^ macrophages as sender cells may be affected by sender-cell abundance, and the lack of detectable SEMA3C–NRP1 signaling in IPF may partly reflect limited cell numbers rather than a complete biological absence of this interaction.

Two recent scRNA-seq studies of explanted RA-ILD lungs reported changes in both immune and stromal compartments (26). One study identified altered immune-cell composition, including changes in FCN1^+^CD14^+^ monocytes, whereas another described transcriptional remodeling of macrophages and fibroblasts in RA-ILD, with some shared fibrotic programs between RA-ILD and IPF (27). These findings are broadly consistent with our observation that myeloid and fibroblast populations are both altered in RA-ILD. Differences in the abundance of FCN1^+^ cells may reflect differences in cohort composition, comparator groups, and cell-state annotation. In our dataset, FCN1^+^ macrophages were enriched in RA-ILD and were associated with SEMA3C–NRP1 signaling and an APOE^+^CXCR4^+^ fibroblast state.

The fibroblast analysis identified a matrix-fibroblast-to-APOE^+^CXCR4^+^ trajectory in the RA-ILD samples, whereas IPF samples mainly accumulated myofibroblasts. This contrast may reflect different inflammatory microenvironments, but pseudotime represents an inferred ordering rather than direct lineage tracing. Future studies combining PPARγ immunofluorescence with lipid-droplet staining will be required to determine whether the APOE^+^ fibroblast cluster identified here corresponds to canonical lipofibroblasts. The CXCL12–CXCR4 axis has context-dependent roles in lung injury, contributing to both profibrotic mesenchymal-cell recruitment and tissue-repair responses (28, 29). In our dataset, CXCR4 expression was concentrated in APOE^+^ inflammation-associated fibroblasts in RA-ILD rather than in the myofibroblast population enriched in IPF, suggesting a fibroblast state distinct from conventional IPF-associated myofibroblasts. However, because extracellular matrix production and reparative functions were not directly assessed, this population cannot yet be classified as definitively profibrotic or reparative.

CellChat further predicted a SEMA3C–NRP1 interaction from inflammatory macrophage populations to matrix fibroblasts, and tissue imaging demonstrated macrophage-associated SEMA3C signal near NRP1^+^ fibroblasts. Neither approach establishes direct ligand transfer or excludes SEMA3C production by other cell types. In particular, the surface-marker-defined sorted population was enriched for inflammatory monocyte/macrophage-lineage cells but was not demonstrated to be a transcriptionally pure FCN1^+^ population.

The *in vitro* experiments provide convergent evidence for a SEMA3C–NRP1-dependent marker response. Patient-derived inflammatory myeloid cells, recombinant SEMA3C, and SEMA3C-overexpressing THP-1-derived macrophages all shifted MRC-5 cells from EBF1/PDGFRA toward APOE/CXCR4, and NRP1 inhibition attenuated this response. These experiments used MRC-5 fetal lung fibroblasts rather than adult or RA-ILD patient-derived primary fibroblasts. Moreover, the endpoints define a fibroblast state associated with inflammation and remodeling; without measurements of COL1A1, fibronectin, α-SMA, matrix deposition or contraction, migration, and proliferation, they do not establish a complete profibrotic functional phenotype.

In summary, this study identifies an FCN1^+^ inflammatory macrophage-associated SEMA3C–NRP1 signaling pattern linked to fibroblast-state remodeling in the RA-ILD samples examined. The small RA-ILD cohort, lack of other CTD-ILD comparators, incomplete post-sort definition of the inflammatory myeloid fraction, use of MRC-5 cells, and marker-focused functional endpoints limit causal and disease-specific interpretation. Accordingly, the proposed axis should be regarded as a candidate mechanism for validation in larger, independent RA-ILD and CTD-ILD cohorts and in adult primary lung fibroblasts.

## Data Availability

The single-cell RNA-sequencing data generated from human lung tissues in this study have been deposited in the Genome Sequence Archive for Human (GSA-Human) at the National Genomics Data Center, China National Center for Bioinformation, under study accession HRA021234 and BioProject accession PRJCA038957. Following release, access to the raw sequencing data will remain controlled in accordance with applicable human-subject privacy and ethical requirements.
