# Supplementary material for: An FCN1+ macrophage-associated SEMA3C–NRP1 axis is linked to fibroblast reprogramming in RA-ILD
Source: Front Immunol. 2026 Oct 23;17:1978823. doi: 10.3389/fimmu.2026.1978823 (PMC13740601; doi:10.3389/fimmu.2026.1978823)
Supplement: Supplementary file 1 [file SupplementaryFile1.docx]

Supplementary Material

# 1 Supplementary Figures

Supplementary Figure 1. Marker expression and annotation of major cell populations and myeloid subtypes.

Figure S1. Myeloid-cell annotation and quality-control analyses.

(a)UMAP plots split by individual samples showing the distribution of major cell populations across adult control, RA-ILD, and pulmonary fibrosis samples. Cell identities are indicated by color.

(b) Re-clustered UMAP of the myeloid compartment showing macrophages, monocytes, and neutrophils.

(c) Dot plot of representative marker genes across neutrophil/granulocyte, monocyte, and macrophage subtypes. Dot size represents the percentage of cells with detectable expression, and color indicates scaled mean expression.

(d) Detection frequencies of selected marker genes in FCN1⁺ monocyte/macrophages and neutrophils.

(e) Distribution of doublet scores across neutrophils, monocytes, and macrophages. The dashed horizontal line indicates the overall 99th-percentile doublet-score threshold.

(f) Myeloid UMAP colored by doublet score.

(g) Distribution of the number of detected neutrophil-core genes per cell among FCGR3B, CSF3R, CXCR2, and MPO in FCN1⁺ monocyte/macrophages and neutrophils.

(h) Neutrophil, monocyte, and macrophage module scores across the three myeloid populations. Black points indicate sample-level median scores.

(i) Myeloid UMAPs colored by neutrophil, monocyte, and macrophage module scores.

Supplementary Figure 2. Subclustering analysis of macrophages, and GO enrichment of FCN1⁺ macrophages.

(a) Feature plots showing representative marker genes across macrophage subsets in single-cell RNA-seq data.

(b–c) GO enrichment analysis of FCN1⁺ macrophages comparing RA-ILD with HCs (b) and IPF (c). GO-term P values were corrected using the Benjamini–Hochberg method; FDR (q) < 0.05 was considered significant.

(d) Positive fluorescence area (%) of COL1A2, CD68, and FCN1 in lung tissue sections from the control, IPF, and RA-ILD groups.

(e) Positive fluorescence area (%) of COL1A2, CD68, and SPP1 in the corresponding groups. Five independent samples were analyzed per group (n = 5). Each dot represents one biological replicate, and bars indicate mean ± SD. Statistical significance was assessed by one-way ANOVA with multiple-comparison testing. *P < 0.05, **P < 0.01, ***P < 0.001; ns, not significant.

Supplementary Figure 3. Characterization and functional analysis of fibroblast subtypes in RA-ILD.

(a) Feature plots showing representative marker genes across fibroblast populations.

(b) Expression of CXCR4 and EREG across fibroblast subsets, highlighting activation-related transcriptional programs.

(c) Violin plots showing repair and inflammation scores among fibroblast subtypes. Active fibroblasts exhibit higher inflammation and repair scores compared to non-active lipofibroblasts.

(d–e) GO enrichment analysis of lipofibroblast-related genes comparing RA-ILD with HCs (d) and IPF (e). GO-term P values were corrected using the Benjamini–Hochberg method; FDR (q) < 0.05 was considered significant.

(f) Representative immunofluorescence images of APOE, CXCR4, and PDGFRA in control, IPF, and RA-ILD lung tissues.

(g, h) Quantification corresponding to Fig. 3k and Fig. 3l, respectively.

(i) Quantification of APOE-, CXCR4-, and PDGFRA-positive fluorescence areas corresponding to panel f.

Supplementary Figure 4. Validation and interaction analysis of the SEMA3C–NRP1 axis between FCN1⁺ macrophages and fibroblasts in RA-ILD.

(a-c) Bubble plots showing predicted ligand–receptor interactions among macrophages and fibroblast subtypes in HCs (a), RA-ILD (b), and IPF (c) groups, as determined by CellChat analysis. Bubble size indicates p-value significance, and color scale represents communication probability.

(d) Cell–cell communication network showing that the SEMA3C–NRP1 interaction was not detected in the IPF group in this dataset. This within-cohort absence does not establish RA-ILD specificity relative to other CTD-ILDs.

(e) Representative immunohistochemical staining of NRP1 in lung tissues from patients with RA-ILD and IPF. Red arrows indicate areas of positive NRP1 staining.

(f) Quantification of the positive staining area (%) for SEMA3C and CD68 in control, IPF, and RA-ILD lung tissues.

(g) Quantification of the positive fluorescence area (%) for COL1A2, NRP1, and SEMA3C in the three groups.

(h) Representative multiplex immunofluorescence images showing DAPI, FCN1, CD68, and SEMA3C staining in lung tissues from RA-ILD, IPF, and control samples. Enlarged regions are shown on the right.

(i) Quantification of the positive fluorescence area (%) for COL1A2, NRP1, and SEMA3C in the corresponding lung tissue samples. Each dot represents one biological replicate, and bars indicate mean ± SD. Statistical significance was assessed using one-way ANOVA followed by multiple-comparison testing. *P < 0.05, **P < 0.01, ***P < 0.001; ns, not significant.

Supplementary Figure 5. Flow-cytometric gating strategy for isolation of the CD45⁺CD11b⁺HLA-DR⁺CD64⁺CD14⁺CCR2⁺ inflammatory monocyte/macrophage-enriched population from RA-ILD lung tissue.

(a) Representative flow cytometry plots showing the gating strategy used to isolate macrophages from enzymatically digested lung tissues of RA-ILD patients.

(b) Representative immunofluorescence images and quantification showing that recombinant SEMA3C treatment did not significantly alter NRP1 expression in fibroblasts.
